# The Association of Dietary Cholesterol and Fatty Acids with Dyslipidemia in Chinese Metropolitan Men and Women

**DOI:** 10.3390/nu10080961

**Published:** 2018-07-25

**Authors:** Zhenni Zhu, Fan Wu, Ye Lu, Zhengyuan Wang, Jiajie Zang, Huiting Yu, Changyi Guo, Xiaodong Jia, Xianbiao Shen, Gangqiang Ding

**Affiliations:** 1 Division of Health Risk Factors Monitoring and Control, Shanghai Municipal Center for Disease Control and Prevention, 1380 West Zhongshan Road, Shanghai 20036, China; zhuzhenni@scdc.sh.cn (Z.Z.); wufan@scdc.sh.cn (F.W.); wangzhengyuan@scdc.sh.cn (Z.W.); zangjiajie@scdc.sh.cn (J.Z.); guochangyi@scdc.sh.cn (C.G.); jiaxiaodong@scdc.sh.cn (X.J.); 2 Division of Non-communicable Diseases Prevention and Control, Shanghai Municipal Center for Disease Control and Prevention, 1380 West Zhongshan Road, Shanghai 20036, China; luye@scdc.sh.cn; 3 Division of Health information, Shanghai Municipal Center for Disease Control and Prevention, 1380 West Zhongshan Road, Shanghai 20036, China; yuhuiting@scdc.sh.cn; 4 Department of Public Health, Shanghai Baoshan District Center for Disease Control and Prevention, 158 Mingyue Road, Shanghai 201901, China; shenxianbiao@126.com; 5 National Institute for Nutrition and Health, Chinese Center for Disease Control and Prevention, 27 Nanwei Road, Xicheng District, Beijing 100050, China

**Keywords:** dietary cholesterol, dietary fatty acids, serum lipids, dyslipidemia, population-based epidemiology

## Abstract

Background: The associations between dietary cholesterol and fatty acids and serum lipids are controversial. This study is to examine the association of dietary cholesterol and fatty acids with serum lipids and dyslipidemia in Chinese metropolitan male and female adults. Methods: 3850 participants in the Shanghai Diet and Health Survey were investigated during the period 2012–2013. Information was obtained on dietary intake, anthropometric and blood laboratory measurements. Dyslipidemia was determined by US National Cholesterol Education Program Adult Treatment Panel III (NCEP-ATP III). Results: Dietary cholesterol was in line with serum TC, LDL-C and the LDL-C to HDL-C ratio in general and the partial correlation coefficients were 0.64 (95% CI: 0.13–1.15, *p* = 0.015), 0.73 (95% CI: 0.21–1.24, *p* = 0.006) and 0.01 (95% CI: 0.00–0.02, *p* = 0.018), respectively. The partial correlation coefficients were greater in women. Dietary fatty acids were not associated with serum lipids. The highest quintile of dietary cholesterol intake (≥538.0 mg/day) was associated with an approximate 1.6-fold risk for high TC and high HDL-C compared with the lowest quintile (<193.1 mg/day) generally. Conclusions: Dietary cholesterol was associated with serum cholesterol in Chinese metropolitan adults and a higher risk of dyslipidemia was observed at a high level of dietary cholesterol intake. Whether there should be an upper limit on dietary cholesterol in the Chinese population warrants further study.

## 1. Introduction

Cardiovascular disease (CVD) contributes to nearly half of all deaths from non-communicable diseases around the world [1]. The relationship between blood cholesterol and CVD is well-established [2]. Elevated low-density lipoprotein-cholesterol (LDL-C) is a well-known risk factor for the development of cardiovascular events and lowering LDL-C is key to reducing the risk of CVD [3,4,5,6]. Dietary cholesterol is considered to raise serum cholesterol concentrations [7].

However, the associations between dietary cholesterol and CVD are highly controversial [8,9]. Cholesterol is synthesized in the body, and the absorption of additional dietary cholesterol is compensated for by reducing cholesterol synthesis [10]. Increased dietary cholesterol intake results in raising serum cholesterol in hyper-response individuals, while normal responders are not significantly affected by dietary cholesterol [11,12]. Other studies reported the lack of correlation between dietary cholesterol and CVD, i.e., dietary cholesterol increased both LDL-C and high-density lipoprotein-cholesterol (HDL-C) but without altering the LDL-C to HDL-C ratio, a key marker of CVD risk [13]. Thus, there has been considerable interest in studying the relationship between dietary cholesterol intake and CVD risk.

The former well-known dietary cholesterol recommendation was no more than 300 mg/day [14]. However, the Scientific Report of 2015 Dietary Guidelines in the United States eliminated the upper limit for dietary cholesterol intake [15]. One year later, the restriction of dietary cholesterol has been excluded from the new edition of Dietary Guidelines for Chinese [16]. This has raised considerable debates in China since dietary cholesterol used to be considered as a restricted nutrient for the prevention of CVD.

Dietary fatty acids are other major determinants of serum lipid concentrations. In some studies, saturated fatty acids (SFAs) were considered as dietary risk factors of CVD and unsaturated fatty acids were considered as preventive factors [17]. However, the associations between dietary fatty acids and the risk of CVD are inconsistent between studies which has led to extensive debates [18,19].

Given the inconsistencies among studies, the primary aim of this study was to determine the association of dietary cholesterol and fatty acids and serum cholesterol and triglyceride concentrations in our Chinese metropolitan male and female adults. In addition, we attempt to examine the association of dietary cholesterol and fatty acids with dyslipidemia and therefore seek information on which to base an appropriate upper limit of dietary cholesterol intake for the Chinese population.

## 2. Materials and Methods

### 2.1. Study Population

Data for this analysis are from a cross-sectional investigation, the baseline survey of Shanghai Diet and Health Survey (SDHS). This reporting is in line with STROBE (Table A1). SDHS is a prospective cohort study investigating the associations between food consumption, energy and nutrients intake, behavioral factors, and nutrition-related health outcomes in adults. This cohort enrolled 4504 community-dwelling men and women aged 18 and above from 54 randomly stratified-sampled communities in the area of Shanghai, China during the period 2012–2013. Participants were initially recruited from a random representative sample of the local population aged 18 and above (*n* = 1725), and then their family members aged 18 and above were recruited accordingly (approximately 2.6 persons per family). Those who had lived locally for less than 6 months in total during the last year of the survey were excluded from the cohort. Shanghai Municipal Center for Diseases Control and Prevention was responsible for the implementation of SDHS.

Participants whose blood samples were not collected or tested (*n* = 508) were excluded, as well as those who reported energy intakes less than 300 kcal/d or greater than 3500 kcal/d (*n* = 51). Participants who missed the dietary survey (*n* = 38) or other pertinent covariates (*n* = 57) were also excluded. The data of 3850 participants were finally included in the present analysis.

SDHS was approved by the Shanghai Municipal Center for Diseases Control and Prevention’s Institutional Review Board; informed consent was obtained by each participant before the survey; and all procedures were carried out in accordance with Institutional Review Board regulations.

### 2.2. Dietary Assessment

The dietary survey included a consecutive 3-day, 24-h diet record (including 2 weekdays and 1 weekend day), and household condiments weighed before and after 3 survey days. Interviewers were public health doctors from 54 local community health centers who received a standard training course to record diet information. Each participant was face-to-face interviewed by interviewers at home. Participants were instructed not to change their typical diet or physical activity during the survey period. Diet records were reviewed by nutrition specialists from local centers of disease control and prevention. There was no disastrous event which might affect normal food supply in the survey period.

Daily food consumption was calculated from a 3-day, 24-h diet recall record. The 3-day condiments consumption from condiments weighing in a household was divided into individual intake according to times of eating at home and individuals’ energy proportion among family members. Dietary energy, macronutrients, cholesterol and fatty acids intake was estimated according to the daily food and condiments consumption by using the Chinese food composition database [20,21].

### 2.3. Potential Confounders

Participants’ age, sex, education, income, smoking status and physical activity level were ascertained by an interviewer-administered questionnaire at their homes. The educational level of the participants was reported in years of education. The yearly income was calculated by dividing total family yearly income by the number of family members. The physical activity level was recorded as sedentary, moderate and vigorous according to professional and non-professional activity level. Smoking status was categorized as never smoker, former smoker and current smoker. Anthropometric measurements were conducted in the community health centers local to the participants. Body mass index (BMI; kg/m^2^) was calculated from measured weight (kilograms) and height (meters).

### 2.4. Laboratory Measurements

Blood was collected from participants after fasting more than 10 h and analyzed at the laboratory of Shanghai Municipal Center for Disease Control and Prevention. Serum concentrations of total cholesterol (TC), LDL-C, HDL-C and triglycerides were all directly tested using a HITACHI 7080 Automatic Biochemical Analyzer with reagents from Wako Pure Chemical Industries, Ltd. (Tokyo, Japan). The ratio of LDL-C to HDL-C (LDL-C: HDL-C) was an indicator for the assessment of individuals at risk for CVD and calculated by dividing LDL-C by HDL-C [22]. The ratio of TC to HDL-C (TC: HDL-C) implied that diet-induced decreases in HDL-C increase CVD risk and was calculated by dividing TC by HDL-C [23].

### 2.5. Definition of Dyslipidaemia

Dyslipidemia was determined according to the guideline of the US National Cholesterol Education Program Adult Treatment Panel III (NCEP-ATP III) [3]. High serum TC was defined as TC ≥ 240 mg/dL; high serum LDL-C was defined as LDL-C ≥ 160 mg/dL; low serum HDL-C was defined as HDL-C < 40 mg/dL in men or < 50 mg/dL in women; high serum triglycerides was defined as triglycerides ≥ 150 mg/dL.

### 2.6. Statistical Analyses

Statistical analyses were conducted using SAS statistical software (v. 9.2; SAS Institute, Cary, NC, USA). Differences between male and female in the characteristics of participants were examined using Chi-square test and Student’s t test. Considering that serum indicators were possibly independent in family members which may cause aggregation bias, multilevel models were used to determine the ICCs (Intra-class Correlation Coefficient: within-family variance divided by total variance) of the dependent variables (serum indicators). After calculation, all ICCs of dependent variables in the present analyses were <0.1 which meant less than 10% total variance of the dependent variables attributed to each family cluster. This result accorded with the factor of small family size (2.6 persons per family) in the current study. Thus, fixed effect models were used to analyze the correlations between dietary intake and serum indicators. Multivariate general linear regression models were used to calculate the correlation coefficients and 95% confidence intervals (CI) between dietary cholesterol and fatty acids intake as the independent variables and serum cholesterol and triglycerides level as the dependent variables. Dietary cholesterol was transformed to portions of 100 g and fatty acids to portions of 10 g. Multivariate binomial logistic regression models were performed to determine the odds ratios (OR) and 95% confidence intervals (CI) of four types of dyslipidemia by five tertiles of dietary cholesterol intake. Potential confounders, including age, sex, years of education, yearly income, smoking status, physical activity level and BMI, were screened using stepwise selection with SLE (selection of entry) = 0.5 and SLS (selection of stay) = 0.10 in both multivariate linear regression and binomial logistic regression analyses. Finally, age, sex, smoking status and BMI comprised the models. To avoid multicollinearity, other dietary factors were not included as covariates. A 2-sided *p* < 0.05 was considered statistically significant.

## 3. Results

### 3.1. Characteristics of Participants

The analysis sample included 3850 participants, of which there were 1838 male adults and 2012 female adults. The proportion of age did not differ between sex. Years of education, smoking status, physical activity level, dietary intake and serum lipids profiles were significantly different between sex (*p* < 0.001). Men had more years of education, proportion of smoking behavior and heavier physical activity level. Women consumed less dietary cholesterol and fatty acids (*p* < 0.001) and had greater levels of serum cholesterol (*p* < 0.001) compared with men. (Table 1)

### 3.2. Linear Regression between Dietary Cholesterol and Serum Cholesterol

Dietary cholesterol was statistically significant in line with serum TC, LDL-C and the LDL-C to HDL-C ratio after adjusting to sex, age, smoking statuses and BMI in all participants. The partial correlation coefficients (β) mean an increase (mg/dL) of serum cholesterol or triglycerides with each additional 100 mg dietary cholesterol intake or 10 g dietary fatty acids intake. In all, the partial correlation coefficients between dietary cholesterol and serum TC, LDL-C and the LDL-C to HDL-C ratio were 0.64 (95% CI: 0.13–1.15, *p* for trend = 0.015), 0.73 (95% CI: 0.21–1.24, *p* for trend = 0.006) and 0.01 (95% CI: 0.00–0.02, *p* for trend = 0.018), respectively. In females, the partial correlation coefficients were greater between dietary cholesterol and TC as well as LDL-C. In males, there was no statistically significant linear trend between dietary cholesterol and serum cholesterol concentrations. We did not find a linear trend between dietary fatty acids and serum cholesterol in the participants. We did not observe that dietary cholesterol and fatty acids were associated with serum triglyceride levels in our study population. (Table 2).

### 3.3. Quintiles of Dietary Cholesterol Intake and Dyslipidemia Risk

Table 3 shows that the highest quintile of dietary cholesterol intake (≥538.0 mg/day) was observed as having an approximate 1.6-fold risk for high TC (*p* = 0.044) and high HDL-C (*p* = 0.030) compared with the lowest quintile (<193.1 mg/day) in general. Consuming ≥538.0 mg dietary cholesterol daily correlated with an increased risk of high LDL-C by 1.8 times compared with the lowest quintile (<193.1 mg/day) in women. The risks of dyslipidemia were not statistically significantly different between the highest and the lowest quintile of dietary cholesterol intake in men.

## 4. Discussion

Numerous studies have reported that dietary cholesterol was associated with an increase in both serum TC and LDL-C and also with the increased LDL-C to HDL-C ratio [8,24,25,26]. In the current study, we found consistent outcomes, revealing that dietary cholesterol intake was positively linear with serum TC, LDL-C and the LDL-C to HDL-C ratio. Our result was also in line with the study focusing on the Chinese elderly population which showed that serum TC and LDL-C were associated with dietary cholesterol and the response was linear [27]. However, it was reported that dietary cholesterol intake did not have an association with risk of CVD in the Asian population [28]. There was a possible explanation for the inconsistency that a lower intake of dietary cholesterol did not induce the risk of CVD [29]. We found a higher average daily intake of dietary cholesterol in the Chinese metropolitan population which exceeded the previous recommendation of no more than 300 mg/day. This was similar to the average daily intake of cholesterol in western countries which tended to be up to 300 mg/day or more [30,31]. However, the daily intakes of cholesterol in other Asian studies were well below that which was previously recommended [28,32,33]. It was reported that dietary cholesterol was shown to increase both LDL and HDL without altering the LDL-C to HDL-C ratio [13]. However, we did not find any correlation between dietary cholesterol and serum HDL-C. Our findings coincided with the results of a study focusing on the Chinese population [27]. A few studies have suggested that an increase in dietary cholesterol of 100 mg/day is associated with increased circulating TC and LDL-C by 1 to 3 mg/dL [8,24,27,34,35]. In our results, increases of 0.6–1.0 mg/dL in serum TC and LDL-C concentrations were observed by each additional 100 mg/day intake of dietary cholesterol. A meta-analysis showed that dietary cholesterol did not statistically significantly change serum triglycerides [8]. Likewise, in our results, no relationship was observed between dietary cholesterol and serum triglycerides.

Previous studies have shown that saturated fatty acids are major determinants of blood cholesterol concentrations and that unsaturated fatty acids have an important role in the prevention of CVD [36,37,38]. However, some recent analyses have provided a different picture, i.e., total fat and types of fat, including saturated and unsaturated fats, were not associated with an increased risk of CVD [18,39,40]. In our observation, we did not find that dietary saturated fatty acids or unsaturated fatty acids were significantly associated with the change of serum cholesterol concentrations or triglycerides concentrations. In fact, the intake of fatty acids in the Chinese population has reached a historically high level [17]. This suggested that the nature of the relationship was more complex than previously assumed and there is probably a plateau in serum response to high dietary fatty acids intake.

Dyslipidemia represents an important determinant of CVD risk [5]. Chinese adults currently experience a high prevalence of abnormal serum lipid levels [41]. Previous observations have shown a plateau in serum cholesterol concentrations correlates with increased dietary cholesterol intake, which implied that excessive intake of dietary cholesterol might not associated with the risk of dyslipidemia [42,43]. Moreover, removal of the dietary cholesterol restriction raised intense arguments in China. Whether another proper dietary cholesterol intake level should be implemented to maintain a healthy population is a new question. In this study, we found that the highest dietary cholesterol intake (≥538.0 mg/day) subgroup had a more than 1.5-fold risk in occurrence of high TC and high LDL-C compared with the lowest intake (<193.1 mg/day) subgroup. This indicated that unlimited dietary cholesterol intake correlated with the risk of dyslipidemia.

It was reported that sex differences in CVD risk existed between men and women in the European studies [44]. Men were found more strongly associated with CVD risk compared with women in a Nordic study [45]. Another European study found that the responses of serum cholesterol to dietary cholesterol did not differ between men and women [46]. In the present study, we found sex differences in the relationship between serum cholesterol and dietary cholesterol. Inconsistent with previous studies, our study showed that compared with men, dietary cholesterol was observed more significant in the association with serum cholesterol and dyslipidemia in women. It was reported that ethnic disparities existed in plasma lipoprotein levels [47]. This inconsistency is probably explained by the ethnic difference between Caucasian and Chinese in the sex-specific serum cholesterol responses to dietary cholesterol.

A limitation of this study is the methodology used to assess dietary intake. We used 3-day, 24-h dietary recall records to obtain food consumption information which was further generated into cholesterol and fatty acids intake based on Chinese food composition. The estimates of dietary cholesterol and fatty acids intake were limited by the accuracy of the participants’ recall. Another limitation is that although we adjusted for a number of potential confounding factors, we still cannot avoid the possibility of recall bias, unresponsive bias, and other unknown confounding factors, which might influence the result of the risk factor analysis. The third limitation is that *trans* fat is one of the major determinants of blood cholesterol concentrations. Due to the incomplete *trans* fat information in the current Chinese food composition database, it was not included in the present analysis of the associations between dietary factors and serum lipids. Finally, the cross-sectional nature of our study does not allow us to evaluate causal associations between dietary cholesterol and fatty acids and dyslipidemia. Therefore, prospective observational studies or trials are expected to clarify the causal relationship between dietary cholesterol and fatty acids and serum lipids.

## 5. Conclusions

We found that dietary cholesterol intake was associated with serum cholesterol in Chinese metropolitan adults. A higher risk of dyslipidemia was observed at a high level of dietary cholesterol intake. Women were more susceptible than men to having an association of dietary cholesterol with serum cholesterol and dyslipidemia. Whether there should be an upper limit on dietary cholesterol intake to maintain health in the Chinese population warrants further study.

## Figures and Tables

**Table 1:** Characteristics of participants by sex in SDHS 2012–2013.

	All	Male	Female	*p-*Value
*n* (%)	3850 (100.0)	1838 (47.7)	2012 (52.3)	
Age, %				0.513
18–44 years	31.3	30.7	32.0	
45–49 years	35.4	35.2	35.6	
60 years	33.3	34.1	32.5	
Ethnicity, %				0.050
Hans (ethnic majority)	99.5	99.7	99.2	
Other minorities	0.5	0.3	0.8	
Years of education, %				<0.001
≤9 years	53.9	49.4	58.1	
9–12 years	23.9	26.1	21.9	
>12 years	22.2	24.6	20.0	
Yearly Income,%				0.523
Above average level (>60,000 RMB)	7.8	7.3	8.2	
Average level (30,000–59,999 RMB)	30.9	32.2	29.9	
Below average level (<30,000 RMB)	56.8	55.8	57.7	
No answer	4.5	4.8	4.2	
Smoking Status, %				<0.001
Never smoker	71.0	41.0	98.8	
Former smoker	5.4	10.8	0.4	
Current smoker	23.6	48.3	0.8	
Physical Activity Level, %				<0.001
Sedentary	84.5	79.4	89.0	
Moderate	13.6	17.5	10.1	
Vigorous	2.0	3.1	0.9	
Dietary intake				
Energy, kcal/day (SE ^1^)	1679.0 (9.2)	1826.2 (13.3)	1542.9 (12.1)	<0.0001
Carbohydrate, g/day (SE)	198.0 (1.3)	212.4 (1.9)	184.6 (1.7)	<0.0001
Protein, g/day (SE)	64.0 (0.4)	69.3 (0.7)	59.1 (0.6)	<0.0001
Total fat, g/day (SE)	71.5 (0.6)	77.9 (0.9)	65.8 (0.8)	<0.0001
SFAs ^1^, g/day (SE)	15.4 (0.1)	16.7 (0.2)	14.2 (0.2)	<0.0001
MUFAs ^1^, g/day (SE)	26.0 (0.2)	28.6 (0.4)	23.7 (0.3)	<0.0001
PUFAs ^1^, g/day (SE)	20.9 (0.2)	22.6 (0.4)	19.3 (0.3)	<0.0001
Dietary cholesterol, mg/day (SE)	377.8 (3.5)	399.1 (5.1)	358.0 (4.8)	<0.0001
BMI ^1^ (kg/m^2^), %				<0.001
Underweight (<18.5)	4.4	3.8	4.9	
Normal weight (18.5–23.9)	49.5	45.1	53.6	
Overweight (24.0–27.9)	35.2	39.9	30.8	
Obese (≥28.0)	11.0	11.2	10.8	
Serum lipids profile				
TC, mg/dL (SE) ^1^	182.9 (0.6)	179.1 (0.8)	186.3 (0.8)	<0.001
LDL-C, mg/dL (SE) ^1^	112.2 (0.6)	111.5 (0.8)	112.9 (0.8)	<0.001
HDL-C, mg/dL (SE) ^1^	56.5 (0.3)	53.5 (0.4)	59.2 (0.4)	<0.001
Triglycerides, mg/dL (SE)	133.5 (1.8)	145.8 (2.9)	122.1 (2.3)	<0.001
Dyslipidemia ^2^				
High TC, %	5.8	4.0	7.4	<0.001
High LDL-C, %	7.6	6.4	8.6	0.027
Low HDL-C, %	20.9	14.8	26.3	<0.001
High triglycerides, %	27.3	31.3	23.6	<0.001

^1^ SE, standard errors; SFAs, saturated fatty acids; MUFAs, monounsaturated fatty acids; PUFAs, polyunsaturated fatty acids; TC, total cholesterol; LDL-C, low-density lipoprotein-cholesterol; HDL-C, high-density lipoprotein-cholesterol; BMI, Body mass index. ^2^ Dyslipidemia was determined as high TC: serum TC ≥ 240 mg/dL; high LDL-C: serum LDL ≥ 160 mg/dL; low HDL-C: serum HDL-C < 40 mg/dL in men or < 50 mg/dL in women; high triglycerides: serum triglycerides ≥150 mg/dL.

**Table 2:** General linear regression between dietary cholesterol and fatty acids intake and serum cholesterol and triglycerides level in SDHS 2012–2013 ^1^.

	**TC**			**LDL-C**			**HDL-C**		
	**β ^2^**	**95% CI**	***p*-Value for Trend**	**β**	**95% CI**	***p*-Value for Trend**	**β**	**95% CI**	***p*-Value for Trend**
**All**									
Cholesterol	0.64	(0.13, 1.15)	0.015	0.73	(0.21,1.24)	0.006	0.15	(−0.08, 0.38)	0.213
SFAs	0.81	(−0.68, 2.29)	0.287	0.37	(−1.17, 1.90)	0.641	0.51	(−0.18, 1.19)	0.145
MUFAs	0.53	(−0.28, 1.35)	0.200	0.07	(−0.77, 0.92)	0.863	0.32	(−0.05, 0.70)	0.093
PUFAs	0.06	(−0.74, 0.85)	0.892	0.35	(−0.47, 1.16)	0.403	0.33	(−0.03, 0.69)	0.075
**Male**									
Cholesterol	0.27	(−0.45, 1.00)	0.459	0.43	(−0.29, 1.16)	0.239	0.00	(−0.32, 0.32)	0.995
SFAs	0.94	(−1.06, 2.95)	0.357	0.80	(−1.29, 2.88)	0.454	0.32	(−0.61, 1.25)	0.501
MUFAs	0.65	(−0.44, 1.73)	0.242	0.45	(−0.67, 1.57)	0.431	0.29	(−0.22, 0.79)	0.264
PUFAs	0.48	(−0.58, 1.53)	0.375	0.71	(−0.37, 1.79)	0.199	0.34	(−0.14, 0.82)	0.169
**Female**									
Cholesterol	0.97	(0.25, 1.68)	0.008	0.94	(0.22, 1.67)	0.011	0.35	(0.02, 0.67)	0.036
SFAs	0.45	(−1.74, 2.63)	0.689	−0.44	(−2.67, 1.80)	0.701	0.85	(−0.14, 1.84)	0.094
MUFAs	0.33	(−0.88, 1.55)	0.593	−0.49	(−1.75, 0.77)	0.445	0.41	(−0.15, 0.97)	0.147
PUFAs	−0.45	(−1.64, 0.75)	0.463	−0.18	(−1.40, 1.04)	0.775	0.33	(−0.21, 0.88)	0.227
	**LDL-C:HDL-C**			**TC:HDL-C**			**Triglycerides**		
	**β**	**95% CI**	***p*-Value for Trend**	**β**	**95% CI**	***p*-Value for Trend**	**β**	**95% CI**	***p*-Value for Trend**
**All**									
Cholesterol	0.01	(0.00, 0.02)	0.018	0.01	(−0.01, 0.03)	0.304	0.77	(−0.90, 2.45)	0.364
SFA	0.00	(−0.04, 0.03)	0.801	−0.03	(−0.08, 0.02)	0.242	−2.49	(−7.34, 2.36)	0.314
MUFA	−0.01	(−0.03, 0.01)	0.318	−0.02	(−0.04, 0.01)	0.226	−0.73	(−3.38, 1.92)	0.589
PUFA	−0.01	(−0.02, 0.01)	0.513	−0.02	(−0.05, 0.00)	0.092	−1.88	(−4.48, 0.72)	0.156
**Male**									
Cholesterol	0.01	(−0.01, 0.03)	0.206	0.01	(−0.02, 0.03)	0.632	0.38	(−2.18, 2.94)	0.771
SFA	0.00	(−0.04, 0.05)	0.888	−0.03	(−0.10, 0.04)	0.441	−3.91	(−11.01, 3.19)	0.280
MUFA	0.00	(−0.03, 0.02)	0.810	−0.02	(−0.05, 0.02)	0.435	−1.52	(−5.36, 2.32)	0.437
PUFA	0.00	(−0.03, 0.02)	0.897	−0.02	(−0.05, 0.02)	0.416	−1.41	(−5.15, 2.33)	0.460
**Female**									
Cholesterol	0.01	(0.00, 0.03)	0.093	0.01	(−0.02, 0.03)	0.527	0.93	(−1.19, 3.06)	0.390
SFA	−0.02	(−0.07, 0.02)	0.304	−0.04	(−0.11, 0.02)	0.201	−1.70	(−8.16, 4.75)	0.605
MUFA	−0.02	(−0.05, 0.00)	0.114	−0.02	(−0.06, 0.02)	0.248	0.04	(−3.55, 3.63)	0.984
PUFA	−0.01	(−0.04, 0.01)	0.303	−0.03	(−0.07, 0.00)	0.075	−2.25	(−5.78, 1.28)	0.211

^1^ General linear regression models were used to estimate β and 95% CIs adjusted by age (continuous), sex (male, female), BMI (continuous) and smoking statuses (never smoker, former smoker, current smoker). When stratified by sex, models were adjusted by age, BMI and smoking statuses. ^2^ β represents the partial correlation coefficients in the model, which means the increase (mg/dL) of serum cholesterol or triglycerides by each additional 100 mg dietary cholesterol intake or 10 g dietary fatty acids intake.

**Table 3:** ORs (95% CI) for dyslipidemia according to the quintiles of dietary cholesterol intake (mg/day) in SDHS 2012–2013 ^1^.

		Quintiles of Dietary Cholesterol Intake (mg/day)				
		Q1 (<193.1)	Q2 (193.1–293.0)	Q3 (293.1–401.6)	Q4 (401.7–529.9)	Q5 (≥538.0)
**All (*n* = 3850)**						
*n*		770	770	770	770	770
High TC	OR	Reference	1.19	1.33	1.14	1.57
	95% CI		(0.76, 1.87)	(0.85, 2.08)	(0.71, 1.81)	(1.01, 2.43)
	*p*-Value		0.448	0.210	0.585	0.044
High LDL-C	OR	Reference	0.98	1.04	1.10	1.59
	95% CI		(0.63, 1.54)	(0.66, 1.64)	(0.70, 1.74)	(1.05, 2.42)
	*p*-Value		0.931	0.858	0.666	0.030
Low HDL-C	OR	Reference	0.99	0.85	1.03	0.83
	95% CI		(0.75, 1.30)	(0.64, 1.13)	(0.78, 1.36)	(0.62, 1.11)
	*p*-Value		0.914	0.256	0.843	0.207
High triglyceride	OR	Reference	1.05	0.88	1.04	1.21
	95% CI		(0.83, 1.33)	(0.69, 1.12)	(0.82, 1.32)	(0.96, 1.53)
	*p*-Value		0.681	0.304	0.741	0.111
**Male (*n* = 1838)**						
*n*		316	342	359	409	412
High TC	OR	Reference	1.47	1.40	0.73	1.51
	95% CI		(0.64, 3.34)	(0.61, 3.17)	(0.29, 1.82)	(0.69, 3.32)
	*p*-Value		0.361	0.426	0.494	0.305
High LDL-C	OR	Reference	0.90	1.00	0.81	1.27
	95% CI		(0.43, 1.87)	(0.49, 2.03)	(0.39, 1.69)	(0.66, 2.45)
	*p*-Value		0.777	0.992	0.583	0.478
Low HDL-C	OR	Reference	1.03	0.85	0.73	0.82
	95% CI		(0.65, 1.65)	(0.53, 1.39)	(0.45, 1.19)	(0.52, 1.31)
	*p*-Value		0.892	0.525	0.202	0.411
High triglyceride	OR	Reference	1.38	1.01	1.15	1.33
	95% CI		(0.97, 1.97)	(0.70, 1.44)	(0.81, 1.62)	(0.95, 1.87)
	*p*-Value		0.072	0.978	0.436	0.100
**Female (*n* = 2012)**						
*n*		454	428	411	361	358
High TC	OR	Reference	1.05	1.31	1.38	1.56
	95% CI		(0.61, 1.81)	(0.77, 2.25)	(0.80, 2.37)	(0.91, 2.67)
	*p*-Value		0.855	0.322	0.247	0.104
High LDL-C	OR	Reference	1.00	1.05	1.33	1.84
	95% CI		(0.56, 1.77)	(0.58, 1.89)	(0.75, 2.38)	(1.07, 3.16)
	*p*-Value		0.998	0.882	0.332	0.027
Low HDL-C	OR	Reference	0.96	0.86	1.29	0.82
	95% CI		(0.68, 1.35)	(0.60, 1.23)	(0.91, 1.83)	(0.57, 1.19)
	*p*-Value		0.815	0.414	0.157	0.303
High triglyceride	OR	Reference	0.84	0.83	1.01	1.18
	95% CI		(0.60, 1.16)	(0.59, 1.16)	(0.72, 1.41)	(0.85, 1.65)
	*p*-Value		0.287	0.272	0.969	0.328

^1^ Multivariate binomial logistic regression models were used to estimate ORs and 95% CIs adjusted by age (continuous), sex (male, female), BMI (continuous) and smoking statuses (never smoker, former smoker, current smoker). When stratified by sex, models were adjusted by age, BMI and smoking statuses.

## Appendix A

**Table A1:** STROBE Checklist—cross-sectional studies.

	Item No	Recommendation	Check Notes by the Author
**Title and abstract**	1	(a) Indicate the study’s design with a commonly used term in the title or the abstract	(a) A descriptive statement was revised instead of the previous definitive one.
		(b) Provide in the abstract an informative and balanced summary of what was done and what was found	(b) Conclusive statements in the abstract were revised to use appropriate language for observational studies.
		**Introduction**	**Introduction**
Background/rationale	2	Explain the scientific background and rationale for the investigation being reported	Background and rationale were clearly explained.
Objectives	3	State specific objectives, including any prespecified hypotheses	Objectives were stated. A sentence was revised according to appropriate describing of observational studies.
		**Methods**	**Methods**
Study design	4	Present key elements of study design early in the paper	Clearly stated the design of present study.
Setting	5	Describe the setting, locations, and relevant dates, including periods of recruitment, exposure, follow-up, and data collection	Described the setting, locations, and relevant dates. Described there was no settings that might affect the dietary intake or nutritional status of the participants.
Participants	6	(a) Give the eligibility criteria, and the sources and methods of selection of participants	(a) The criteria and methods of selection of participants were reported.
Variables	7	Clearly define all outcomes, exposures, predictors, potential confounders, and effect modifiers. Give diagnostic criteria, if applicable	Defined all outcomes, dietary intake, predictors and potential confounders. Dyslipidaemia definition was given.
Data sources/measurement	8 *	For each variable of interest, give sources of data and details of methods of assessment (measurement). Describe comparability of assessment methods if there is more than one group	The sources of data and details of methods of dietary and laboratory measurements were given.
Bias	9	Describe any efforts to address potential sources of bias	Described potential confounders and their assessment.
Study size	10	Explain how the study size was arrived at	Explained the study size and the size of the participants’ data used in current study were given.
Quantitative variables	11	Explain how quantitative variables were handled in the analyses. If applicable, describe which groupings were chosen and why	Explaind the reasonable scope of the quantitative variables (energy intakes) and the data within the scope were chosen.
Statistical methods	12	(a) Describe all statistical methods, including those used to control for confounding	(a) Described all statistical methods, including those used to control for confounding
		(b) Describe any methods used to examine subgroups and interactions	(b) Described methods used to examine subgroups
		(c) Explain how missing data were addressed	(c) Explained in the study population that the records with missing data were excluded.
		(d) If applicable, describe analytical methods taking account of sampling strategy	(d) Described estimation and on ICC considering the sampling and decision on using fixed models.
		(e) Describe any sensitivity analyses	(e) Described covariates selection procedure and the parameters setting.
		**Results**	**Results**
Participants	13 *	(a) Report numbers of individuals at each stage of study—e.g., numbers potentially eligible, examined for eligibility, confirmed eligible, included in the study, completing follow-up, and analysed	(a) Reported numbers of individuals’ in the current analysis. The number of individuals at each stage of study was reported in the methods part.
		(b) Give reasons for non-participation at each stage	(b) Gave reasons for non-participation at each stage in the methods part.
		(c) Consider use of a flow diagram	(c) Not using flow diagram.
Descriptive data	14 *	(a) Give characteristics of study participants (e.g., demographic, clinical, social) and information on exposures and potential confounders	(a) Gave characteristics of study participants and information on potential confounders.
		(b) Indicate number of participants with missing data for each variable of interest	(b) Records with missing data were excluded.
Outcome data	15 *	Report numbers of outcome events or summary measures	Reported serum lipids level and numbers of dyslipidaemia.
Main results	16	(a) Give unadjusted estimates and, if applicable, confounder-adjusted estimates and their precision (e.g., 95% confidence interval). Make clear which confounders were adjusted for and why they were included	(a) Gave confounder-adjusted estimates and their precision (e.g., 95% confidence interval). Stated confounders adjusted for. The covariates selection was explained in the methods part.
		(b) Report category boundaries when continuous variables were categorized	(b) Reported the dietary cholesterol category boundaries when it was categorized in the logistic regression model.
		(c) If relevant, consider translating estimates of relative risk into absolute risk for a meaningful time period	
Other analyses	17	Report other analyses done—e.g., analyses of subgroups and interactions, and sensitivity analyses	Reported the analyses of sex subgroups.
		**Discussion**	**Discussion**
Key results	18	Summarise key results with reference to study objectives	Summarised key results with reference to study objectives.
Limitations	19	Discuss limitations of the study, taking into account sources of potential bias or imprecision. Discuss both direction and magnitude of any potential bias	Discussed four limitations of the current study and potential bias or imprecision.
Interpretation	20	Give a cautious overall interpretation of results considering objectives, limitations, multiplicity of analyses, results from similar studies, and other relevant evidence	Gave a cautious overall interpretation of results in the descriptive statements.
Generalisability	21	Discuss the generalisability (external validity) of the study results	Discussed the consistency and inconsistency with other studies.
		**Other information**	**Other information**
Funding	22	Give the source of funding and the role of the funders for the present study and, if applicable, for the original study on which the present article is based	Gave the source of funding and the role of the funders for the present study.

* Give information separately for exposed and unexposed groups. Note: An Explanation and Elaboration article discusses each checklist item and gives methodological background and published examples of transparent reporting. The STROBE checklist is best used in conjunction with this article (freely available on the Web sites of PLoS Medicine at http://www.plosmedicine.org/, Annals of Internal Medicine at http://www.annals.org/, and Epidemiology at http://www.epidem.com/). Information on the STROBE Initiative is available at www.strobe-statement.org.
